# Maternal satisfaction with breastfeeding in the first month postpartum and associated factors

**DOI:** 10.1186/s13006-020-00312-w

**Published:** 2020-08-17

**Authors:** Andrea Francis Kroll de Senna, Camila Giugliani, Juliana Avilla, Agnes Meire Branco Leria Bizon, Ana Cláudia Magnus Martins, Elsa Regina Justo Giugliani

**Affiliations:** 1 Faculdade de Medicina, Programa de Pós-Graduação em Saúde da Criança e do Adolescente, Universidade Federal do Rio Grande do Sul (UFRGS), Rua Ramiro Barcelos, 2400 – 2° andar, Porto Alegre, RS CEP 90035-003 Brazil; 2 Faculdade de Medicina, Programa de Pós-Graduação em Epidemiologia, Universidade Federal do Rio Grande do Sul (UFRGS), Porto Alegre, RS Brazil

**Keywords:** Breastfeeding, Personal satisfaction, Surveys and questionnaires

## Abstract

**Background:**

Breastfeeding success has been measured based on its duration, disregarding satisfaction with the maternal experience. Studies to investigate maternal satisfaction with breastfeeding are rare, especially in Brazil, and little is known about their determinants. The aim of this study was to measure the level of satisfaction with breastfeeding in a group of women in the first month of their child’s life, and to identify factors associated with higher maternal satisfaction.

**Methods:**

A cross-sectional study nested within a cohort was conducted with 287 women recruited at two (one public, one private) maternity services in the city of Porto Alegre, southern Brazil, from January to July 2016. Women residing in the municipality who had given birth to a healthy singleton born at term, were rooming in, and had initiated breastfeeding were randomly included. During the week after the child was 30 days old, women were interviewed at their homes to measure the level of maternal satisfaction with breastfeeding, using the Maternal Breastfeeding Evaluation Scale (MBFES), validated for use in the Brazilian population. Associations between maternal satisfaction and explanatory variables were estimated using multivariate Poisson regression with robust variance in a four-level hierarchical approach. Satisfaction level was categorized using as cutoff point the median score obtained with the MBFES. Women with scores equal to or above the median were considered to have higher levels of satisfaction, whereas those scoring below the median were considered to be less satisfied.

**Results:**

Maternal satisfaction with breastfeeding in the first month postpartum was high, with a median score of 124 on MBFES, close to the maximum score (145 points). The prevalence of more elevated levels of satisfaction with breastfeeding was higher among women with brown (*pardo*) and black skin color (prevalence ratio [PR] 1.33, 95%CI 1.05;1.69), those who lived with the partner (PR 1.75, 95%CI 1.05;2.94), who planned to breastfeed for 12 months or more (PR 1.48, 95%CI 1.02;2.17), and who did not report low milk supply (PR 1.47, 95%CI 1.03;2.10) or cracked nipples (PR 1.29, 95%CI 1.01;1.65).

**Conclusions:**

The factors associated with maternal satisfaction with breastfeeding in the first month postpartum include individual factors and maternal expectations, family constitution, as well as breastfeeding-related problems.

## Background

Despite the advancement in scientific knowledge related to the benefits of breastfeeding for the health of children and women [1], the incidence and prevalence rates of breastfeeding are low in most parts of the world. A recent WHO commissioned publication, which provided complete information on 127 low and middle-income countries and 37 high-income countries, showed that globally the prevalence of early initiation of breastfeeding is less than 50%, exclusive breastfeeding in children 0–5 months is less than 40% and continued breastfeeding at 12 months, although more widespread in low and middle-income settings, is uncommon in more developed countries [1]. Many efforts have been made to improve this situation, taking into account different aspects involved in the success of breastfeeding: social, economic, cultural, political and individual factors. However, one aspect that may influence the duration of breastfeeding, but has been little explored in the literature, is women’s satisfaction with breastfeeding.

Few studies have investigated the association between maternal satisfaction with breastfeeding and the duration of this practice. More than 20 years ago, Riordan et al. found that women’s satisfaction with breastfeeding was positively associated with their intended duration. Although, in this study, women’s satisfaction with breastfeeding was not directly associated with the length of time the mother actually breastfed, the researchers believed that a strong association between satisfaction and duration of breastfeeding is expected [2] This association was confirmed a few years later by Galvão [3] and Cooke et al. [4], who showed that mothers who were most satisfied with their breastfeeding experience were the ones who breastfed the longest. Also, successful maternal perceptions of breastfeeding showed a strong positive correlation with total breastfeeding duration (r = 0.83; *p* < 0.001), breastfeeding exclusivity (r = 0.63; *p* < 0.001), and age at introduction of non-human milk (r = 0.93; *p* < 0.001) [5].

Even more rare are studies about factors associated with increased breastfeeding satisfaction. Some have been identified, namely: early skin-to-skin contact, encouraging breastfeeding on demand in the maternity ward [6], absence of breastfeeding-related problems [4] and maternal self-esteem [3].

To the authors’ knowledge, no studies have been conducted to measured maternal satisfaction with breastfeeding in the Brazilian population nor to investigate factors associated with this satisfaction. We believe that these data, combined with the knowledge of associated factors, can be useful in the development of strategies aimed at increasing maternal satisfaction with breastfeeding, and eventually, the rates of breastfeeding. Therefore, the aims of this study were to measure the level of satisfaction with breastfeeding in a group of Brazilian women in the first month of child’s life, and to identify factors associated with this outcome.

## Methods

This was a cross-sectional study nested within a cohort involving women who gave birth at two large-scale maternity services in the municipality of Porto Alegre, southern Brazil, one public and one private. The public maternity service is part of a general university hospital accredited by the Baby-Friendly Hospital Initiative. The private maternity service is also located within a general hospital. Both are reference maternity hospitals for usual and high-risk pregnancies and have performed 3725 (public hospital) and 4182 (private hospital) deliveries in 2016, representing 26,1% of all deliveries in the municipality during this year [7].

Sample size was calculated using the Programs for Epidemiologists for Windows (WinPEPI) version 11.43. Considering a significance level of 5%, a power of 90%, an estimated prevalence of satisfaction with breastfeeding ranging from 88 to 96% [8] and a minimum prevalence ratio (PR) of 1.25, a minimum sample size of 277 was calculated for variables with a minimum prevalence of 30%.

Women were selected about 24 h after delivery. Mothers residing in the municipality of Porto Alegre, who were rooming in with their newborns, and had initiated breastfeeding, were included. Newborns should be singletons and have been born at term, and should not have any problems that could interfere with breastfeeding, such as orofacial malformations or conditions that required mother-child separation. Women who lived in especially dangerous areas, where home visits had been restricted by health authorities for security reasons, were not included, as a measure to protect the research team.

Every day, from January to July 2016, two women were selected at the public maternity service and one at the private service, resulting in a sample that was similar to the Brazilian population regarding use of public/private health services: 60 to 70% of Brazilian population use solely the public health system [9–11]. The eligible women were selected by drawing lots performed by one of the researchers. Following selection, the first contact was made with the mothers, and those who agreed to participate were requested to sign an informed consent form. Also at this first contact, a date was set for the interview, which should take place when their child was between 31 and 37 days old, at the woman’s home or a location of her choice, outside the hospital environment. All mothers who started breastfeeding at the maternity hospital were included in this study and visited by the researchers, regardless of having ceased breastfeeding after discharge. The interviews lasted for approximately 60 min and were conducted by 10 previously trained interviewers. A questionnaire was used to obtain data on sociodemographic characteristics, the woman’s health, last pregnancy, labor and peripartum, as well as some aspects of the first month of life of the newborn, e.g. type of feeding. Maternal satisfaction with breastfeeding was measured using the Maternal Breastfeeding Evaluation Scale (MBFES), adapted and validated for use in the Brazilian population [12]. The adapted instrument contains 29 questions on positive and negative experiences and emotions related to breastfeeding, measured using a five-point Likert-type scale ranging from “totally disagree” (1 point) to “totally agree” (5 points); for negative experiences, the scoring system is inverted. Higher scores indicated higher levels of satisfaction. Home visits were made instead of phone calls mainly because the instrument (MBFES Brazil) is self-applicable.

In the first stage of the analysis, 37 variables were selected as possible explanatory variables of the outcome, namely: **maternal characteristics** (age, skin color, schooling, parity, working status when getting pregnant, alcohol use); family characteristics (socioeconomic level, living with partner, paternal schooling); pregnancy characteristics (planning, smoking during pregnancy); **prenatal care characteristics** (number of prenatal care visits, satisfaction with prenatal care); **labor and peripartum characteristics** (public or private hospital, maternal satisfaction with childbirth and rooming in, intended duration of breastfeeding and exclusive breastfeeding, postpartum complications [mother or newborn], newborn’s birth weight and sex, gestational age, type of delivery, skin-to-skin contact immediately after delivery, newborn put to the breast within first hour of life, breastfeeding support in delivery room, breastfeeding support provided by professionals, professional guidance on breastfeeding, rooming in, use of pacifier, use of infant formula, use of nipple shield; **characteristics of the first month** (use of pacifier, use of infant formula or other milk, difficulties with breastfeeding, professional breastfeeding support).

Statistical analyses were performed using the Statistical Package for the Social Sciences (SPSS) version 21.0 (SPSS Inc., Chicago, IL, USA). Associations were estimated using Poisson regression with robust variance. A hierarchical regression model was developed based on Boccolini et al. [13], in which, variables were grouped into hierarchically organized blocks according to their temporal proximity with the outcome [14]. The hierarchical approach is a statistical treatment that allows to evaluate how variables of the same hierarchical group compete with each other, and how more distal variables can mediate the effects of variables in the groups closest to the outcome. In this model, a conceptual framework is built based on knowledge of social and biological determinants, considering the temporal relationship between these and the outcome [14]. Therefore, the variables were distributed into four different blocks or levels: (1) distal, including sociodemographic, maternal, and family characteristics; (2) distal intermediate, including pregnancy characteristics; (3) proximal intermediate, including childbirth and peripartum characteristics; and (4) proximal, including variables related to the child’s first month of life.

At first, analyses were conducted to examine the association between the outcome and each of the variables assessed in each block. Variables in the distal block reaching a significance level of *p* < 0.20 in the univariate analysis were subjected to Poisson multivariate regression (intrablock analysis). Subsequently, variables in the distal intermediate block that reached *p* < 0.20 in the univariate analyses were subjected to Poisson multivariate regression along with the distal block variables that reached *p* < 0.10 in the multivariate analysis; and so on. The model predicted that variables reaching *p* < 0.10 in the intrablock analysis should remain in the model until the end, as confounders, adjusting interactions between variables from the different blocks. The association between the different variables assessed and the outcome was estimated using prevalence ratio (PR) and 95% confidence intervals (95%CI). Statistical significance was set at *p* < 0.05.

The level of maternal satisfaction with breastfeeding was categorized using as cutoff point the median score obtained with the MBFES. Women with scores equal to or above the median were considered to have higher levels of satisfaction, whereas those scoring below the median were considered to be less satisfied. Explanatory variables were categorized as dichotomous, except for maternal age and years of schooling. Birth weight was categorized according to the median obtained.

This project was approved by the ethics committees of the maternity hospitals involved.

## Results

Of the 354 women initially included, 287 completed the study (194 from the public and 93 from the private maternity service) due to losses (18.9%), i.e., were not found for the interview at 30 days, after at least three contact attempts via telephone calls and home visits in the first month after childbirth.

The 67 women lost to follow-up were similar to those who completed the study in terms of type of delivery, parity, and infant’s sex, but had fewer years of schooling (*p* < 0.01) and showed a higher prevalence of white skin color (*p* = 0.032).

Sample characteristics are shown in Table 1. Mean maternal age was 29 ± 6.6 years, ranging from 16 to 45 years; the 20–34 age group was the most prevalent one (69.3%). Most women (71%) had completed at least secondary education (11 years or more of schooling) and belonged to socioeconomic level B according to the classification [15] (41.4%). The sample was homogeneous in terms of type of delivery and parity.

**Table 1:** Results of univariate Poisson regression analysis for variables selected for inclusion in the multivariate model

Variables	Total (***n*** = 287)		Higher satisfaction levels		PR (95%CI)	p
Distal block	n	%	n	%		
Age (years)*						0.168
Mean (SD)	29.08 (6.60)				0.99 (0.97,1.00)	
**Skin color**						0.011
Brown (*pardo*)/black	71	24.7	45	63.4	1.34 (1.07,1.68)	
White	216	75.3	102	47.2	1	
**Socioeconomic level**^a^						0.024
A	45	15.8	15	33.3	0.61 (0.40,0.94)	
B/C/D/E	240	84.2	131	54.6	1	
Years of schooling – father’s child*^b^						0.017
Mean (SD)	12.55 (4.23)				0.96 (0.94,0.99)	
**Living with patner**						
Yes	248	86.4	135	54.4	1.77 (1.09,2.87)	0.021
No	39	13.6	12	30.8	1	
**Distal intermediate block**	**n**	**%**	**n**	**%**		
**Planned pregnancy**						0.096
Yes	154	53.7	86	55.8	1.22 (0.96,1.35)	
No	133	46.3	61	45.9	1	
**Proximal intermediate block**	**n**	**%**	**n**	**%**		
**Type of maternity service**						0.109
Public	194	67.6	106	54.6	1.24 (0.95,1.61)	
Private	93	32.4	41	44.1	1	
**Type of delivery**						0.043
Vaginal	149	51.9	85	57.0	1.27 (1.01,1.60)	
Cesarean section	138	48.1	62	44.9	1	
**Breastfeeding support in delivery room**^**c**^						0.152
Yes	136	47.9	75	55.1	1.18 (0.94,1.49)	
No	148	52.1	69	46.6	1	
**Baby put to breast within first hour of life**^**c**^						0.072
Yes	189	66.5	104	55.0	1.27 (0.98,1.66)	
No	95	33.5	41	43.2	1	
**Breastfeeding support while rooming in**^a^						0.076
Yes	255	89.5	136	53.3	1.60 (0.95,2.69)	
No	30	10.5	10	33.3	1	
**Use of formula at maternity service**^a^						0.051
Yes	94	33.0	40	42.6	0.77 (0.59,1.00)	
No	191	67.0	106	55.5	1	
**Use of nipple shield at maternity service**						0.049
Yes	36	12.5	12	33.3	0.62 (0.38,0.99)	
No	251	87.5	135	53.8	1	
**Intended breastfeeding duration**^d^						0.024
≥ 12 months	192	78.0	105	54.7	1.55 (1.06,2.28)	
< 12 months	54	22.0	19	35.2	1	
**Birth weight (g)**						0.142
≤ 3280	144	50.2	80	55.6	0.84 (0.67,1.06)	
> 3280	143	49.8	67	46.9	1	
**Proximal block**	**n**	**%**	**n**	**%**		
Low milk supply**						< 0.001
No	203	70.7	120	59.1	1.84 (1.32,2.56)	
Yes	84	29.3	27	32.1	1	
Pain while breastfeeding**						0.020
No	105	36.6	63	60.0	1.30 (1.04,1.62)	
Yes	182	63.4	84	46.2	1	
Nipple crack**						0.019
No	152	53.0	88	57.9	1.32 (1.05,1.67)	
Yes	135	47.0	59	43.7	1	
Difficulty latching on**						0.020
No	224	78.0	125	55.8	1.30 (1.04,1.62)	
Yes	63	22.0	22	34.9	1	
Nipple anatomy problems**						0.010
No	228	79.4	126	55.3	1.60 (1.12;2.28)	
Yes	59	20.6	21	35.6	1	
Breast milk oversupply**						0.155
No	129	44.9	60	46.5	0.84 (0.67,1.06)	
Yes	158	29.3	87	55.1	1	
**Use of formula or other milk at 30 days**						< 0.001
No	193	70.2	119	61.7	1.94 (1.42,2.65)	
Yes	82	29.8	27	32.9	1	
**Use of pacifier at 30 days**						0.007
No	88	30.7	55	62.5	1.35 (1.08,1.68)	
Yes	199	69.3	92	46.2	1	

*PR* Prevalence ratio, *95%CI* 95% confidence interval, *SD* Standard deviation

^*^ Variables continuous

^* *^ All difficulties with breastfeeding were reported at the interview

^a^ Social classes (A, B1, B2, C1, C2, D-E) according to ABEP, 2015^15^. Missing values: 2

^b^ Missing values: 16

^c^ Missing values: 3

^d^ 41 did not know

The prevalence of children leaving the maternity hospital being exclusively breastfed was 81.9% and at the end of the first month, 61.7%; and the prevalence of any breastfeeding in these time points were 98,3% and 95,8%, respectively. MBFES scores in the first month postpartum ranged from 63 to 145, with a median of 124 and a mean score of 120 ± 14. The 25th percentile cutoff score of the sample was 113 and in the 75th percentile was 131 points.

Table 1 also presents the results of the univariate analysis of the variables selected for inclusion in the multivariate model (*p* < 0.2).

**Fig. 1:**
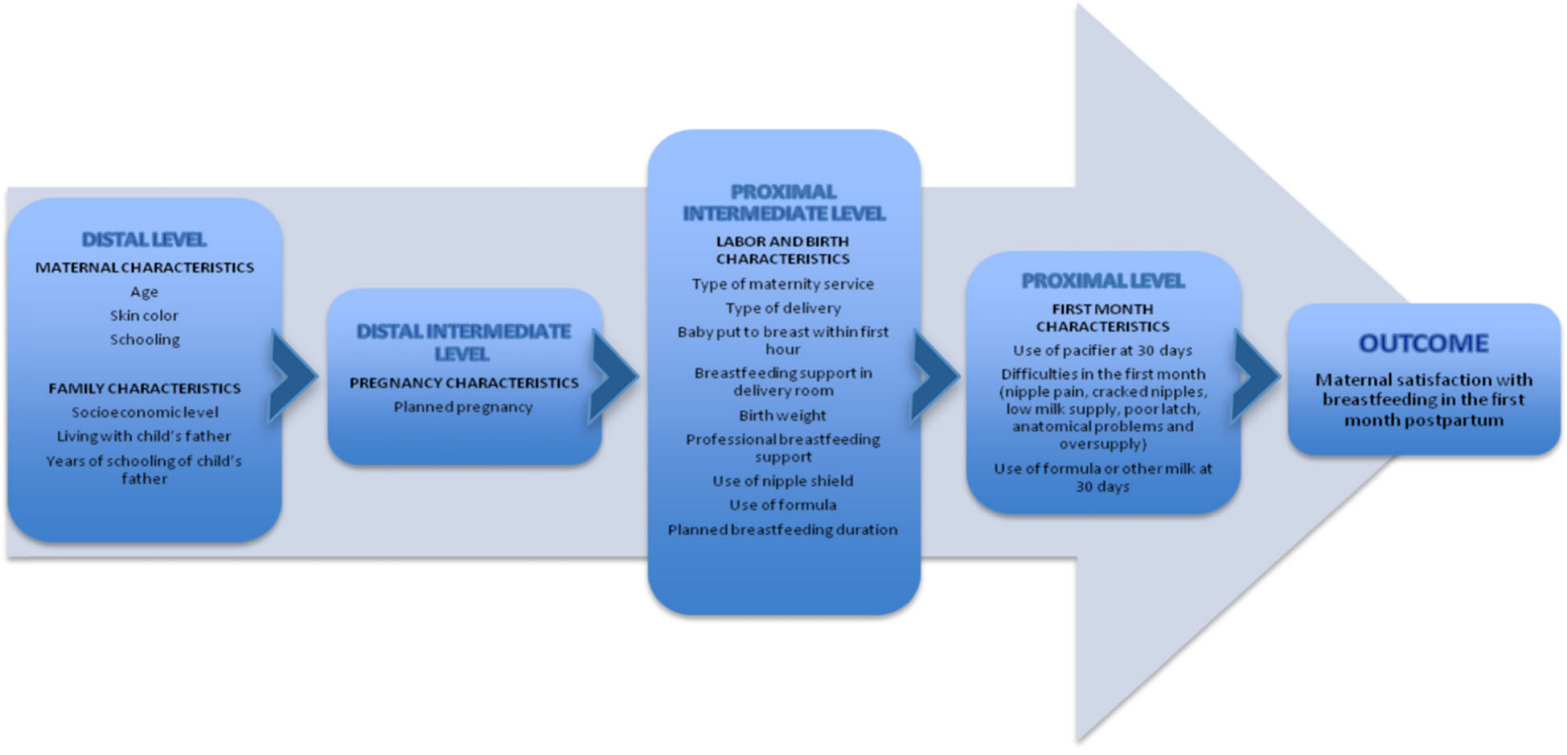
Hierarchical model for the analysis of factors associated with maternal satisfaction with breastfeeding

Fig. 1 shows the hierarchical model used for the multivariate analysis and Table 2 the results of this analysis. At the distal level, women with brown (*pardo*) and black skin color (PR 1.33, 95%CI 1.05;1.69) and those who lived with partner (PR 1.75, 95%CI 1.05;2.94) showed higher levels of satisfaction with breastfeeding. At the distal intermediate level, the only variable included in the model was not associated with the outcome. At the proximal intermediate level, women who planned to breastfeed for 12 months or more were more satisfied (PR 1.48, 95%CI 1.02;2.17). Finally, at the proximal level, women who did not report low milk supply or cracked nipples showed higher satisfaction (PR 1.47, 95%CI 1.03;2.10; and PR 1.29, 95%CI 1.01;1.65, respectively).

**Table 2:** Factors associated with higher levels of maternal satisfaction with breastfeeding in the first month

Variables	Distal block^*^		Distal intermediate block^**^		Proximal intermediate block^***^		Proximal block^****^	
	(model 1)		(model 2)		(model 3)		(model 4)	
	PRa (95%CI)	p	PRa (I95%CI)	p	PRa (95%CI)	p	PRa (95%CI)	p
**Age (years)**^a^	0.99 (0.98,1.02)	0.951						
**Skin color**		0.018						
Brown (*pardo*)/black	**1.33 (1.05,1.69)**							
White	1							
**Socioeconomic level**		0.142						
A	0.69 (0.42,1.13)							
B/C/D/E	1							
**Living with partner**		0.033						
Yes	**1.75 (1.05,2.94)**							
No	1							
**Years of schooling – child’s father**^a^	0.99 (0.95,1.02)	0.429						
**Planned pregnancy**				0.374				
Yes			1.11 (0.88,1.41)					
No			1					
**Type of maternity service**						0.474		
Public					0.88 (0.63,1.24)			
Private					1			
**Type of delivery**						0.079		
Vaginal					1.30 (0.97,1.75)			
Cesarean section					1			
**Breastfeeding support in delivery room**						0.585		
Yes					0.92 (0.67,1.25)			
No					1			
**Baby put to breast within first hour of life**						0.478		
Yes					1.14 (0.80,1.63)			
No					1			
**Breastfeeding support while rooming in**						0.323		
Yes					1.30 (0.78,2.14)			
No					1			
**Use of formula at maternity service**						0.117		
Yes					0.78 (0.58,1.06)			
No					1			
**Use of nipple shield at maternity service**						0.161		
Yes					0.70 (0.43,1.15)			
No					1			
**Intended breastfeeding duration**						0.041		
≥ 12 months					**1.48 (1.02,2.17)**			
< 12 months					1			
**Birth weight (g)**						0.293		
≤ 3280					1.13 (0.90,1.43)			
> 3280					1			
**Low milk supply** ^b^								0.034
No							**1.47 (1.03,2.10)**	
Yes							1	
**Pain while breastfeeding** ^b^								0.949
No							0.99 (0.78,1.26)	
Yes							1	
**Nipple crack**^b^								0.043
No							**1.29 (1.01,1.65)**	
Yes							1	
**Difficulty latching on**^b^								0.105
No							1.37 (0.94,2.02)	
Yes							1	
**Nipple anatomy problems**^b^								0.383
No							1.17 (0.82,1.65)	
Yes							1	
**Breast milk oversupply**^b^								0.436
No							1.10 (0.87,1.38)	
Yes							1	
**Use of formula or other milk at 30 days**								0.163
No							0.77 (0.54,1.11)	
Yes							1	
**Use of pacifier at 30 days**								0.819
No							0.97 (0.77,1.23)	
Yes							1	

*PRa* adjusted prevalence ratio; *95%CI* 95% confidence interval, *SD* Standard deviation

Women with higher levels of satisfaction: MBFES score ≥ median (124)

^*^n = 270

^**^n = 287

^***^n = 240

^****^n = 246

^a^ Variables analyzed as continuous

^b^ All difficulties with breastfeeding were reported at the interview

## Discussion

The present study revealed a high level of maternal satisfaction with breastfeeding in the first month postpartum, considering that the median MBFES score obtained (124 points) was close to the maximum score (145 points). In a study conducted in Australia, the mean scores obtained with the MBFES for maternal satisfaction with breastfeeding were 116 at 15 days postpartum, 117 at 45 days, and 120 at 3 months. The authors did not report median scores, but did report terciles: < 110, 110–126, and > 126 [4]. Taking into consideration that the instrument validated for the Brazilian population had one less item compared to the original instrument, reducing the maximum score by 5 points, it is possible to conclude that, overall, the levels of maternal satisfaction with breastfeeding were higher in our sample when compared to the Australian study, as our median was closer to the cutoff point adopted for the highest satisfaction tercile in that sample. In France, another study found percentages of women highly satisfied and satisfied with breastfeeding at 6 months ranging from 88 to 96%, depending on the maternity service analyzed. However, those authors did not use an instrument to measure satisfaction; rather, the outcome was assessed using one single question inquiring about the level of maternal satisfaction with the breastfeeding experience; response options were very unsatisfactory, unsatisfactory, satisfactory, and very satisfactory [8]. A similarly simple approach to the measurement of maternal satisfaction with breastfeeding at 6 months was also recently adopted in Norway, where the investigators found high levels of satisfaction in over 75% of the women assessed [16].

This study identified five variables that showed significant association with higher satisfaction levels with breastfeeding: women’s brown/black skin color, living with partner, having planned to breastfeed for at least 12 months, not reporting low milk supply, and not reporting cracked nipples in the first month. It is important to highlight that these associations have not been described previously, except for the association between satisfaction and breastfeeding-related problems [3, 4, 8].

The variable most strongly associated with higher levels of maternal satisfaction with breastfeeding in this study was living with partner. Women who lived with their partners, which comprised the majority of the sample, showed a 75% higher prevalence of increased satisfaction, defined in this study as having a score equal to or above the median MBFES score. It is known that having a support network is a determining factor for the breastfeeding experience to be considered positive by the mother [17]. In this sense, probably the presence of the partner may be a positive influence, probably by reassuring the mother and providing support to the different demands that emerge as the child is born, making the experience more pleasurable for the mother. Indeed, several studies have demonstrated the importance of the partner for several breastfeeding-related outcomes, especially breastfeeding duration [17–20]. Nevertheless, no study to date has specifically assessed the aspect of the father or partner on maternal satisfaction with breastfeeding.

Women with brown and black skin color showed a higher prevalence of increased satisfaction with breastfeeding (PR 1.33). This result may be related to the fact that, in Brazil, black and brown-skinned women breastfeed for longer than white-skinned ones – median of 14.6 months vs. 10.1 months, respectively [21]. This result was confirmed in the most recent Brazilian nation-wide survey – infants with black skin colour aged 6 to 24 months had the highest rates of breastfeeding [22]. The differences of behavior among races are usually attributed to customs, norms and social traditions [23] as well as income [24] and social relations [25].

Another variable associated with higher levels of satisfaction was the intended duration of breastfeeding. Women who reported wanting to breastfeed for at least 12 months showed a higher prevalence of increased satisfaction. These women probably gave more value to breastfeeding and were more informed and aware of the importance of this practice. It is possible that the women who intended to breastfeed for shorter periods, despite the universal recommendation to breastfeed for 2 years or more [26], for some reason did not have the same willingness to breastfeed as those who planned to breastfeed for longer, which may have effects on their satisfaction levels. Our study is the first to describe this association. However, Labarère et al., in France, had already found an association between higher levels of satisfaction with breastfeeding at 6 months and agreement between intended and actual breastfeeding duration [8].

Women who did not report low milk supply or cracked nipples in the first month showed higher levels of satisfaction with breastfeeding in our study. The association between breastfeeding problems and satisfaction with breastfeeding had been described previously [3, 4, 8]. Labarère et al. study, found that facing any breastfeeding difficulty after discharge was independently associated with lower levels of satisfaction [8]. Cooke et al., using MBFES in Australia, also observed that perceived low milk supply in the first 2 weeks postpartum was negatively associated with satisfaction with breastfeeding, and that, at 6 weeks, breastfeeding problems reduced satisfaction levels significantly [4]. In a Portuguese study, also using MBFES, women who did not have breastfeeding difficulties in the maternity ward reported higher levels of satisfaction; this association was highly significant at 3 months, but lost significance at 6 and 12 months [3]. Therefore, not having breastfeeding-related problems, especially in the beginning, seems to favor positive feelings towards the breastfeeding experience. Conversely, an American study assessing a convenience sample of 30 first-time breastfeeding mothers had that satisfaction with breastfeeding as measured by the MBFES 1 week after birth could be present regardless of the occurrence of breastfeeding problems or the quality of the experience [27].

The present study did not confirm associations reported previously between satisfaction with breastfeeding and maternal age [5], early skin-to-skin contact, encouraging breastfeeding on demand in the maternity ward [6], and receiving breastfeeding guidance in the maternity ward [3]. The fact that, in our study, maternal satisfaction with breastfeeding was not related to maternal age, parity, socioeconomic level, type of delivery, breastfeeding promotion practices, or pacifier use deserves to be highlighted, as all these factors are described in the literature as associated with breastfeeding duration [13]. This suggests that the determinants of breastfeeding duration do not seem to be the same as of breastfeeding satisfaction. It is also worth noting that the level of satisfaction of the women who were exclusively breastfeeding their children at 30 days of life was not different from that of women who were already offering infant formula at this time (since this variable did not fulfill the criterion to enter the multivariate analysis model), showing that satisfaction with breastfeeding does not seem to depend on compliance with the recommendation to exclusively breastfeed infants in the first months of life.

This is the first study to assess maternal satisfaction with breastfeeding in Brazil using an instrument validated for this purpose. It is also the first to investigate possible determinants of this satisfaction, exploring a wide variety of factors. In addition to the innovative character of the study, another strength was the hierarchical multivariate analysis employed, which allowed the evaluation of the interaction between variables within the same group, and how more distal variables could mediate the effects of variables in groups that are closer to the outcome.

On the other hand, the study has limitations, such as the number of participants below the minimum calculated for variables with prevalence below 30%. However, the study supports low PR (1.25) for the majority of investigated risk factors and the hierarchical model adds power to the multivariate model, as the number of variables reduces when the analyzes are made by blocks. Another limitation of the study was the non-inclusion in the sample of women who resided in areas considered to be too dangerous for home visits; this may have affected the external validity of the study. As a result, the present findings can be generalized only to populations with a similar profile to the sample here assessed, mainly because the breastfeeding process can be influenced by multiple factors (individual, environmental, emotional, sociocultural). It is expected that the level of satisfaction with breastfeeding and associated factors will vary across populations. Thus, further studies with larger samples and in different populations with distinctive characteristics should be done in order to better understand the multiple factors involved in the maternal satisfaction with breastfeeding.

## Conclusions

In conclusion, the present study showed that the factors associated with maternal satisfaction with breastfeeding in the first month postpartum in the population assessed included individual factors and maternal expectations, family constitution, as well as breastfeeding-related problems. These factors do not necessarily overlap with the determinants of breastfeeding duration, as several variables classically associated with breastfeeding duration did not show association with satisfaction at 1 month. We believe that working to increase maternal satisfaction with breastfeeding should be a part of breastfeeding promotion strategies. Efforts in this direction should initiate during prenatal care, with special attention to the fact that women who plan to breastfeed for less than 12 months and who do not live with partner may tend to have lower levels of satisfaction, as here suggested. Also, prevention of breastfeeding problems seems to be an important strategy to increase maternal satisfaction with breastfeeding. The assumption that a woman who is satisfied with breastfeeding may influence other women to adopt the same practice, makes this approach to satisfaction with breastfeeding even more relevant. Therefore, strategies to increase women’s satisfaction with breastfeeding should be incorporated into programs of breastfeeding promotion. The addition of satisfaction to the already known determinations of breastfeeding is an important aspect to be considered as a suggestion emerging from this study.

## Data Availability

The datasets used and analyzed during the current study are available from the corresponding author on reasonable request.

## Abbreviations

CI: Confidence interval
MBFES: Maternal Breastfeeding Evaluation Scale
PR: Prevalence ratio
SPSS: Statistical Package for the Social Sciences
WinPEPI: Programs for Epidemiologists for Windows
